# The material and biological characteristics of osteoinductive calcium phosphate ceramics

**DOI:** 10.1093/rb/rbx024

**Published:** 2017-09-08

**Authors:** Zhurong Tang, Xiangfeng Li, Yanfei Tan, Hongsong Fan, Xingdong Zhang

**Affiliations:** National Engineering Research Center for Biomaterials, Sichuan University, Chengdu 610064, P.R. China

**Keywords:** calcium phosphate ceramics, porous structure, osteoinduction, tissue regeneration

## Abstract

The discovery of osteoinductivity of calcium phosphate (Ca-P) ceramics has set an enduring paradigm of conferring biological regenerative activity to materials with carefully designed structural characteristics. The unique phase composition and porous structural features of osteoinductive Ca-P ceramics allow it to interact with signaling molecules and extracellular matrices in the host system, creating a local environment conducive to new bone formation. Mounting evidence now indicate that the osteoinductive activity of Ca-P ceramics is linked to their physicochemical and three-dimensional structural properties. Inspired by this conceptual breakthrough, many laboratories have shown that other materials can be also enticed to join the rank of tissue-inducing biomaterials, and besides the bones, other tissues such as cartilage, nerves and blood vessels were also regenerated with the assistance of biomaterials. Here, we give a brief historical recount about the discovery of the osteoinductivity of Ca-P ceramics, summarize the underlying material factors and biological characteristics, and discuss the mechanism of osteoinduction concerning protein adsorption, and the interaction with different types of cells, and the involvement of the vascular and immune systems.

## Introduction

Advances in material and life sciences have brought modern medicine to the brink of innovative abilities to repair and replace damaged tissues or organs. From simply repairing the physical shape to regenerating a living tissue or organ, from large-scale surgery wounds to minimally invasive repair, the traditional non-living biomaterials are facing challenges. A promising frontier in this area priority-developing frontier is to take advantage of the body’s natural ability to regenerate, especially to endow biomaterials with the biological function to induce tissue or organ regeneration. To achieve this aim, growth factors and cultured cells were incorporated into the biomaterials to realize the biological function of inducing tissue regeneration. However, the high cost of the growth factors, the time-consuming process of cell extraction and *in vitro* expansion, the immunological rejection, as well as the difficulty in transportation and storage of the living cells or growth factors, hindered the development of tissue regeneration. Therefore, materials with the ability to induce tissue formation possessed high potential for tissue regeneration.

Calcium phosphate (Ca-P) ceramics are extensively regarded as excellent bone grafts due to their good biocompatibility, osteoconductivity [1, 2]. However, it was not demonstrated that Ca-P ceramics could induce bone formation until the end of 1980s. In 1988, Heughebaert [3] reported the bone-like substance induced by hydroxyapatite (HA) ceramics in soft tissue of hamsters. In 1991, the initial histological evidences of the osteoinduction of Ca-P ceramics were shown by Ripamonti [4] and our group [5]. These studies indicated that it is possible to endow biomaterials with osteoinductive ability by optimizing the material characteristics rather than by adding living cells or growth factors to induce tissue regeneration.

The discovery of materials osteoinduction has highlighted the potential to explore new generation of biomaterials; therefore, a large number of publications have extensively studied the osteoinductivity of Ca-P and other materials, and a preliminary theory of this kind of osteoinduction has been established. More promisingly, a new concept of tissue-inducing biomaterials has been brought up based on the osteoinduction of materials. In this review, we will summarize the osteoinduction of Ca-P on the point of material factors affecting osteoinduction, biological characteristics and processes related to osteoinduction, as well as the mechanism of osteoinduction initiated by materials. And finally, a perspective view for the development of tissue-inducing materials will be given.

## The material characteristics of osteoinductive materials

Of all materials that are currently used in clinics for bone implants and grafts, Ca-P ceramics holds the greatest promise to be developed into true bone-replacing material owing to the similarity of its chemical composition to bone minerals as well as its biocompatibility, osteoconductivity and osteoinductivity [5–9]. A wealth of studies has now firmly linked the osteoinductivity of Ca-P ceramics to myriad material factors that can be optimized in the fabrication process [10–12]. Generally, a three-dimensional (3D) porous structure with bone-like apatite surface layer and certain structural characteristics intrinsic to Ca-P ceramics is crucial to new bone induction [7, 10–15]. In the ensuing paragraphs, we will discuss in detail the effects of various material characteristics of Ca-P materials on osteoinductivity.

### Phase composition and solubility

A number of studies about material factors related to osteoinductivity of Ca-P ceramics have demonstrated that the phase composition is one of the most important factors in inducing bone formation [16, 17]. Many groups used ‘chemical compositions’ to describe this concept, but we prefer ‘phase composition’ instead because Ca-P biomaterials with same chemical composition may assume different crystal phases such as α- and β-tricalcium phosphate (TCP). Classified by phase composition, the widely referred Ca-P materials include dicalcium phosphate dihydrate (DCPD), dicalcium phosphate anhydrous (DCPA), HA, biphasic calcium phosphate (BCP, both HA/α-TCP and HA/β-TCP), tricalcium phosphate (α- and β-TCP) and octacalcium phosphate (OCP) [7–9, 15–17]. Table 1 shows the Ca/P ratio and aqueous solubility (*K*_sp_) of several Ca-P materials [18–20].

**Table 1.** Ca/P ratios and the aqueous solubility of several Ca-P phases (25 °C) [18–20]

Ca-P phase	Formula	Ca/P ratio	Solubility (*K*_sp_)
DCPA	CaHPO_4_	1	1.87 × 10^−7^
DCPD	CaHPO_4_·H_2_O	1	2.59 × 10^−7^
HA	Ca_10_(PO_4_)_6_(OH)_2_	1.67	6.62 × 10^−126^
α-TCP	Ca_3_(PO_4_)_2_	1.5	8.46 × 10^−32^
β-TCP	Ca_3_(PO_4_)_2_	1.5	2.07 × 10^−33^
OCP	Ca_8_H_2_(PO_4_)_6_·5H_2_O	1.33	1.01 × 10^−94^

Among the Ca-P materials, HA, β-TCP and BCP (HA/β-TCP) have been studied extensively for their osteoinducing activity [7–9, 16]. HA is the most stable and least soluble one (*K*_sp_≈6.62 × 10^−126^), and was occasionally found to achieve osteoinductivity due to its low dissolution rate. β-TCP possesses a Ca/P ratio of 1.5 and *K*_s__*p*_ value of 2.07 × 10^−33^ at 25 °C, which is more soluble than HA. Although β-TCP has a higher dissolution rate, it is difficult to retain a temporary mechanical support for the desired duration of time [15]. Therefore, a composite mixture consisting of poorly soluble HA and highly soluble β-TCP with different β-TCP/HA ratios was regarded to be able to achieve an optimum surface solubility. Based on the reported results, the trend of osteoinductivity can be ordered as BCP > β-TCP > HA ≫ α-TCP [7–9, 15, 17, 21]. The number of reports about osteoinduction of BCP is the highest, then HA and β-TCP, while few reports referred the osteoinduction in α-TCP [17]. In the early studies, most reported Ca-P osteoinduction were found in BCP although the actual ratio of HA and TCP was vague. In our recent study [8], we compared the osteoinductivity of BCP ceramics with various mass ratios of β-TCP/HA, and found that BCP consisting of 30% HA and 70% β-TCP promoted higher expression of bone morphogenetic protein-2 (BMP-2) and showed stronger osteoinductivity in mice than BCP (70% HA and 30% β-TCP), pure β-TCP and HA.

As materials with different phase compositions have different solubility, phase composition is partly attributed to the varied solubility of the Ca-P ceramics. From the above phase composition and solubility data, as well as the comparison of osteoinductivity, it seems that a higher solubility of Ca-P ceramics would result in higher osteoinductivity, such as that in BCP and HA ceramics. However, it does not always happen in this way. For example, β-TCP ceramic has a relatively high solubility than BCP ceramic, while its osteoinductivity is not superior to BCP ceramics, according to several studies [7–9, 17]. Furthermore, the osteoinductivity was occasionally observed in some non-ceramic Ca-P materials such as Ca-P cements [22–24]. This obviously weaker osteoinductivity was partly due to the lack of porous structure and the unstable interface although they might be more soluble than Ca-P ceramics.

Based on the comparison of Ca-P with different phase compositions, a general conclusion can be inferred that with similar porous structure, BCP ceramic with higher solubility is much more osteoinductive than HA ceramic with lower solubility.

### Ca^2+^ and ${\text{PO}}_{4}^{3}$^−^ ionic releasing

Due to the fact that the osteoinductive phenomenon is usually observed in Ca-P ceramics and the varied osteoinductivity with Ca-P phase composition and solubility, calcium (Ca^2+^) and phosphate ions (${\text{PO}}_{4}^{3}$^−^) are suspected to be of great importance for material osteoinduction. Another clue comes from the fact that during *in vivo* bone resorption, osteoclasts are likely to release Ca^2+^ and ${\text{PO}}_{4}^{3}$^−^, derived from bone matrix**.** This causes a local increase in the ion concentration to supersaturating levels, which has a significant impact on the proliferation and differentiation of osteoblasts, as well as on the subsequent bone formation process [25].

In fact, Ca^2+^ gradients are observed in many extracellular microenvironments and regarded as potent chemical signals for cell migration and directed growth [25, 26]. Additionally, Ca^2+^ is an important homing signal, which brings together different types of cells required for the initiation of bone remodeling [25, 27]. For instance, high Ca^2+^ concentrations are showed to stimulate pre-osteoblastic chemotaxis to the site of bone resorption, and their maturation into cells that produce new bone [25, 28]. On the other hand, the release of extracellular Ca^2+^ also plays an important role in controlling the proliferation and differentiation of osteoblasts near the bone resorption site [25, 29]. Similarly, ${\text{PO}}_{4}^{3}$^−^ also appears to play an important role in osteoinduction. Moreover, ${\text{PO}}_{4}^{3}$^−^ is believed to play a critical role in physiological bone matrix mineralization [25, 30].

In addition, the ionic environment might initiate bone induction by affecting protein adsorption. For example, in one of our works, we found that the solubility of Ca-P ceramics (β-TCP > BCP > HA, list in Table 1) also influenced protein adsorption by affecting the equilibrium ion concentration near the material surface [31]. We also found higher adsorption of fibrinogen, insulin and type I collagen (COL-I) on BCP than that on HA surfaces [31, 32]. Here, we speculated that the release of Ca^2+^ and ${\text{PO}}_{4}^{3}$^−^ from more soluble β-TCP phase in BCP caused a local increase in ion concentration, thus resulting in more Ca-P precipitation, hence promoted protein adsorption, which in turn promoted better osteogenesis. Furthermore, these studies may also explain why BCP ceramics, with a mixture of highly soluble and poorly soluble phases, perform better than either HA or TCP in promoting osteoblastic differentiation.

### Macro- and micro-porous structure

Apart from the effects of phase composition and Ca^2+^/${\text{PO}}_{4}^{3}$^−^ ionic environment, the pore structures have been shown to play an important role in the osteoinductivity of Ca-P ceramics. Almost all the reported osteoinductive materials have an interconnected porous structure. Our experiments have confirmed that porous Ca-P ceramics can induce bone formation while dense Ca-P ceramics cannot [10, 33]. The basic function of pores inside the scaffold is to accommodate ingrown cells, while the role of the pore interconnected channel is to allow body fluid, blood vessels, and cells to develop toward the center of the scaffold, as well as the adequate exchange of oxygen and nutrition [7, 9, 10, 13, 33, 34]. In the case of osteoinductive materials, blood vessels also have the additional function of bringing along cells with the capacity to differentiate into osteoblasts [34].

The porous structural parameters of scaffolds include porosity, pore size, pore shape and pore connectivity [7, 9]. High porosity is believed to create significantly positive effects in terms of enhancing osteogenesis, but the positive effect does not necessarily take place because the low strength arising from an overly high porosity often causes untimely collapse of the implanted scaffold [9]. Therefore, for bioactive ceramic scaffolds, a porosity ranging from 40% to 80% is available for bone defect repair, depending on the implanted sites. In addition, the number of open pores is also directly related to bone formation. The interconnected pores and passing pores look like inner tunnels allowing mass transport, cell adhesion and bone ingrowths [7, 9, 35, 36]. Habibovic et al. observed that after implanting bulk cement of DCPA that contained channels, bone was mainly formed in the interior of the peripheral channels, close to their openings, after remaining for 12 weeks in the muscle of goats [24]. For a given porosity ranging from 40% to 80%, an optimal pore diameter is generally within the range of 200–500 μm, and the pore-interconnected channel size is in the range of 100–200 μm [7, 9].

Besides that, micro-pores (pore diameter < 10 μm) play a crucial role in promoting osteogenesis. The micro-pores on the walls of macro-pores are not only beneficial to penetrating bodily fluids, but also produce rough surfaces on the walls, which are favorable for cell attachment and the expression of osteogenic phenotype [7, 9, 10, 16, 17, 33]. The internal pores yet confine the flow of bodily fluid, thus keeping the concentration of dissolved Ca^2+^ and ${\text{PO}}_{4}^{3}$^−^ ion in the pores and decreasing the shear stresses exerted on the cells and proteins attached on macro-pore surfaces [33]. In one of our works, we found that HA and BCP particles with high porosity and/or more pores >20 nm in size adsorbed more fibrinogen and insulin than particles with low porosities [31]. We further suggested that the distribution of micro-pores on the walls of macro-pores might play a positive role in favoring the adsorption of proteins with low molecular weights (e.g. β1 transforming growth factor, TGF-β1) [37].

**Figure 1.**
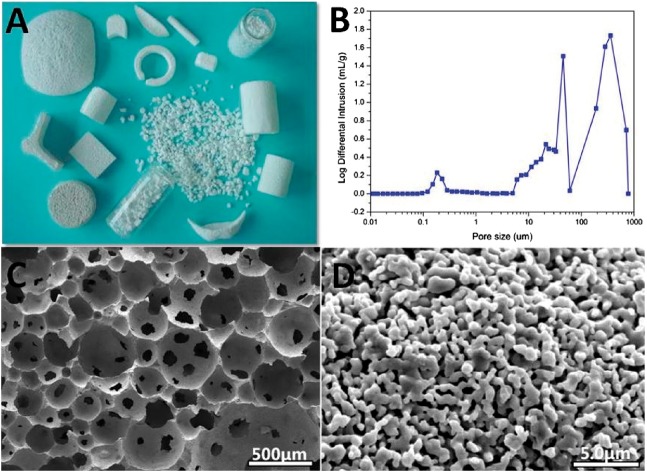
Pore structure of Ca-P ceramic fabricated by the gas-foaming method. (**A**) Porous Ca-P ceramic scaffolds, (**B**) pore size distribution and (**C** and **D**) macro- and micro-pore structure

In our works, gas-foaming method (hydrogen peroxide as gas-foaming agents) was used to fabricate Ca-P ceramics and osteoinductive Ca-P ceramics were successfully prepared [5, 7–9, 31, 33, 34, 37]. In this foaming module, abundant micro-pores besides interconnecting macro-pores (100 μm) can be formed (shown in Fig. 1). Although the pore size and morphology cannot be controlled rigorously in this process, the porosity can be well controlled at the range of 40–80%, as well as the macro-pores’ diameter at 100–500 μm, with abundant micro-pores (<10 μm).

### Topographical features

Among a variety of material characteristics, the material surface topography seems particularly important to their osteoinductivity, and many reports have explored the effect of material surface topography on the behavior of cell adhesion and differentiation [38–44]. In the study of Dalby et al. [45, 46], they fabricated different kinds of surface nanotopographies, and found that surface topography caused significant changes in mesenchymal stem or stromal cells (MSCs) response. A kind of nanodisplaced topography significantly increased osteospecific differentiation; further study concluded that both symmetric and random nanopit arrays did not induce osteogenic differentiation, while the disordered nanopit pattern induced osteogenic differentiation. This is a typical example of surface topography affecting cell function, which means the material surface topography modulating bone or other tissue formation.

In one of our works, we fabricated several types of surface topography on the HA disc-shaped pellets with various pore sizes and different pore morphologies [47]. The osteoblastic cells’ response on them showed that the macro-pore structure on HA surface favored cell proliferation, while the micro-pore structure up-regulated the early osteoblastic differentiation process. This surface pore structure can be regarded as surface topography, hence the varied cell response represented the effect of topography on cell biofunction and even osteogenetic potential. In another work, we fabricated HA ceramic with an orderly micro-patterned surface varied in groove width [48]. The results showed that the cell response also changed with the micro-patterns, proving the effect of surface topography on cell function.

These studies revealed the possibility of surface topography on cell behavior, even cell osteogenic differentiation. In the Ca-P ceramics, the surface topography might also be reflected in surface microstructure variation, resulting from different crystal sizes by different sintering temperatures.

### Bone-like apatite formation

Many studies suggested that the bone-like apatite forming on the surface of Ca-P ceramics played important roles in bone induction [5, 7, 9, 10, 49]. The bone-like apatite is composed of calcium hydroxyl carbonate apatite and is equivalent in composition and structure to the major inorganic phase of natural bones [50–52]. Thus, bone-like apatite forming on an implanted biomaterial has become a direct criterion for evaluating the biomaterial’s bioactivity, although bioactivity is generally defined to be the ability to form chemical bonds between the implanted biomaterials and host tissues [1, 51, 53, 54]. Various studies have demonstrated that porous Ca-P ceramics with the bone-like layer, or those Ca-P ceramics that could induce the bone-like apatite formation *in vivo*, showed strong osteoinductivity [49, 55]. The formation of a bone-like apatite layer on Ca-P ceramics includes the nucleation, growth and crystallization of Ca-P. For Ca-P precipitation, the nucleation mainly depends on the concentrations of Ca^2+^ and ${\text{PO}}_{4}^{3}$^−^ ions, pH, temperature and surface features of the solid onto which Ca-P will be deposited [54, 56–59].

It has been found that in the aqueous system, almost all of the Ca-P ceramics can form a second nuclear apatite layer by dissolution–reprecipitation *in vitro*. The order of the ability of bone-like apatite formation corresponds to the order of osteoinduction of Ca-P ceramics [49]. The study on the relationship of surface micro/nanostructure and biological function of osteoinductivity in the porous BCP ceramics revealed that varied microstructure of the bone-like apatite also affected the material osteoinductivity [49]. And the formation of the bone-like layer with some special characteristics is necessary for osteoinduction of Ca-P ceramics [49].

### Nanoscale and nanostructure

It is well known that the optimal approach of fabricating artificial bone grafts is biomimicry. Bone apatite is composed of nanosized carbonated Ca-P crystals. However, conventional Ca-P ceramics, despite mimicking in part the bony composition and porous structure, have large size of Ca-P grains at the micro-scale, which is likely to debase the biological properties of the porous Ca-P ceramics [7, 9, 60]. In the view of biomimicry, a nanoscale crystal size might improve the biological properties of a Ca-P ceramic. Many *in vitro* studies did prove this hypothesis. Bone forming cells tend to interact with the nanoscale surface of biomaterials, which can promote adhesion, proliferation and differentiation [7, 9, 61–66].

In one of our works, we successfully fabricated the porous Ca-P nanoceramics using modified co-precipitation synthesis and microwave sintering [7, 66]. These Ca-P nanoceramics absorbed more bone-related proteins and showed selective adsorption to some specific proteins, including bone growth factors. Regarding the effect of different surface microstructures, the BCP ceramic surface with feature sizes <100 nm promoted higher protein adsorption than that with feature sizes >100 nm. In addition, because of the high specific surface area, the nanolevel surface topography, the high surface defects and the interconnecting macro-pores with abundant micro-pores, the Ca-P nanoceramics can effectively initiate and regulate a cascade of cellular activities, thereby resulting in higher *invivo* osteoconductivity and osteoinductivity than the conventional Ca-P ceramics.

**Figure 2.**
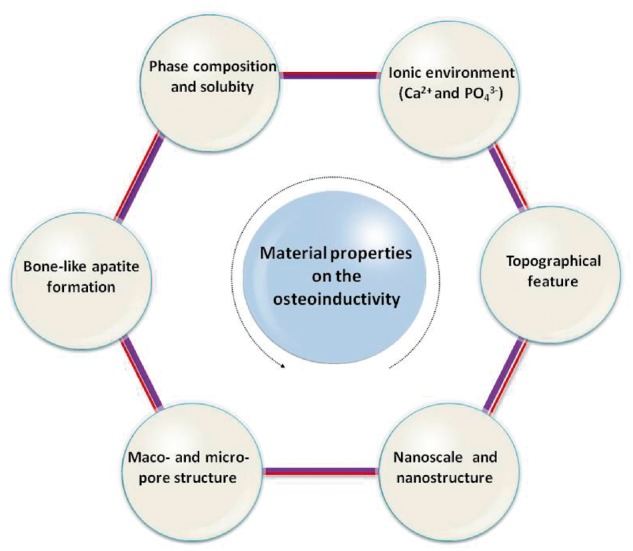
The Material characteristics related to osteoinduction of Ca-P ceramics

In summary, Ca-P ceramics can be endowed with osteoinductivity by optimizing the material characteristics, including phase composition, ionic environment, macro- and micro-pore structure, topographical feature, bone-like apatite and nanostructure. These material properties can influence the process of osteoinduction directly or indirectly (shown in Fig. 2). Although the mechanism of osteoinductivity is still not fully understood, the main material characteristics of Ca-P ceramics relevant to their osteoinductivity are explored. Based on these results, the osteoinductivity of the Ca-P ceramics can be further enhanced by optimizing the physical–chemical properties of materials, and they are also useful for us to gain a deeper and wider understanding of the mechanism for material osteoinductivity.

## Biological characterization of material osteoinduction

The first report of ‘osteoinduction’ came from the works of Urist et al. [67], in which bone formation was observed by implantation of decalcified bone matrix (DBM) or BMPs in muscle of mice, rats, guinea pigs and rabbits. After that, the concept of ‘osteoinduction’ was used for the description of ‘ectopic bone formation’ of biomaterials. In Urist’s experiment, osteoinduction by DBM or BMPs in non-osseous sites were biologically characterized by histological observation. Similar to this phenomenon, some biomaterials such as Ca-P ceramics [68], porous titanium [69] and poly-HEMA [70], were reported to induce ectopic bone formation after implantation in some non-osseous sites, hence demonstrating ‘material osteoinduction’. Normally, *in vivo* implantation of biomaterials in non-osseous sites of animals, followed by a thorough histological analysis and depiction the bone formation, were used to assess the osteoinduction of biomaterials. With the development of bioscience and the extending study of material osteoinduction, more and more characterizing methods and models could be used for exploring material osteoinduction.

### Direct proofs of osteoinduction: histological morphology analysis

Early in 1990s, Zhang, Yamasaki and Ripamonti reported, respectively, the heterotopic bone formation in porous Ca-P ceramics after implantation in dogs and baboons [5, 6, 71]. Later, many following reports gave evidence of Ca-P osteoinduction [10, 14, 15, 36, 68, 72, 73]. In this period, most of the evidence comes from histological staining which revealed morphological evidence of bone tissue. In some cases, TEM or SEM photos were also shown to describe the histological morphology of induced bone or cells [74–76].

Continuous histological observation revealed the process of osteoinduction [10, 13, 72]. Firstly, the fibrous connective tissue and capillaries invaded into the internal pore regions of the Ca-P ceramics. Then the mesenchymal cells arranged around the capillary, ALP-positive cells aggregated at the interface between tissue and biomaterials. After that, osteoblasts were identified. Then, osteoid and bone marrow cavity was formed gradually, and new bone grew from the boundary to the center of the pores. Finally, mature bone with bone marrow cavities formed, and Harvesian system developed in the pores in some cases.

So far, non-osseous or ectopic implantation and histological observations have been well-known standard methods for testifying the osteoinductive ability of biomaterials. By histological analysis, the osteoinduction of different materials, as well as that in different animal species, was extensively studied [13, 68, 75]. In earlier studies, this material osteoinduction was mainly observed in large animals, such as baboons [4], goats [21] and dogs [77]. However, recent works revealed the histological evidence of osteoinduction by Ca-P ceramics in rabbits and mice [73–75, 78].

### Cellular and molecular characterization of material osteoinduction

**Figure 3.**
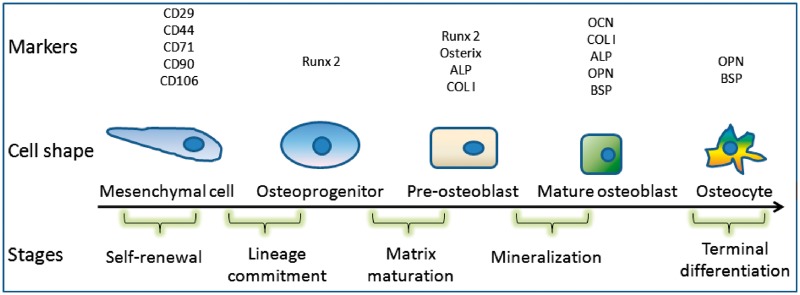
Osteoblastic differentiation of MSCs and markers for different stages

In natural osteogenesis, a series of cellular and molecular events are involved. These events include the osteoblastic differentiation from stem cells to preosteoblasts, osteoblasts and osteocytes, as well as the expression of related bone special markers (genes and proteins) as shown in Fig. 3. As to the new bone formation induced by biomaterials, these cells, genes and proteins should also be presented as evidence, and therefore, characterization of osteogenetic cells and related gene or protein markers has drawn more and more attention in the study of material osteoinduction.

Based on the importance of osteoblastic differentiation of cell in the osteogenesis process, a series of works explored the correlation between material osteoinduction and cell differentiation induced by materials. Although the predication of osteoinduction by *in vitro* culture remains unstable [76], it provides a simplified predictive system for us to understand the mechanism of osteoinduction. These cell models mainly include MSCs and osteoblasts.

#### Mesenchymal stem cells

Stem cells, in particular adult MSCs, with multi-potential differentiation capacity, are currently recognized as a promising cell source for tissue engineering applications and cell-based therapies. The differentiation of MSCs along osteoblastic linage is undoubtedly evidence of osteogenesis. Therefore, identification of MSCs in material osteoinduction indicates the source of potential cells to differentiate into osteoblast, and the differentiation potential influenced by materials might give evidence of material osteoinduction and help to illuminate the possible mechanism.

In searching for MSCs in the osteoinduction of Ca-P, some studies proved the existence of multifunctional cells which might act as MSCs for the osteogenesis inside the materials *in vivo* [72, 79]. We found some multifunctional cells from the samples of Ca-P implanted in muscle [72, 80], which were going through osteoblastic differentiation, indicating the presence of MSCs in the implants and the induction of osteogenesis by Ca-P.

**Figure 4.**
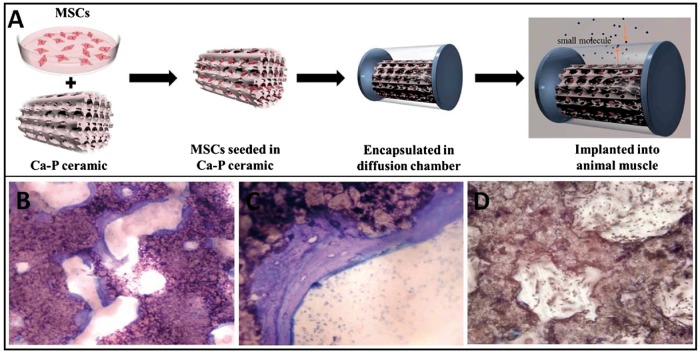
MSCs Isolated from rats’ bone marrow, cultured with Ca-P in a diffusion chamber, then implanted in dogs’ muscle for 6 months (**A**). Histology observation found that bone tissue was formed in the pores of BCP (**B** and **C**), while not in HA (**D**)

Many researchers explored *in vitro* co-culture of MSCs and Ca-P materials, including HA, BCP and TCP, and presented evidence of osteoblastic differentiation of MSCs [81–84]. The consistence between osteoblastic differentiation and osteoinduction was found. In one of our studies, MSCs from rat’s bone marrow (rBMSCs) were co-cultured with BCP in a diffusion chamber and implanted in dog’s back muscle. Half a year later, bone-like tissue was observed (Fig. 4). In this model, the rBMSCs cells were separated from the host tissue by the diffusion chamber, hence the results proved the possibility of materials inducing MSCs’ differentiation along osteoblastic linage and thus forming new bone. In another report, Yuan et al. [82] described an *in vitro* model in which human bone marrow stromal cells (hBMSCs) were co-cultured with four types of Ca-P ceramics, and a trend of gene expression was correlated with the amount of bone induced by the materials implanted intramuscularly in sheep. This indicates that it is possible to predict the osteoinductivity of biomaterials by co-culture of MSCs and biomaterials.

#### Osteoblasts

Osteoblasts are specialized, differentiated products of MSCs. Their function is the synthesis of collagen, and several additional specialized proteins, such as osteocalcin (OCN) and osteopontin (OPN), which compose the organic matrix of bone. Besides MSCs, osteoblasts are also widely used to study the influence of Ca-P materials on cell response, such as their attachment, proliferation and secreting function. Many cell lines, including MC3T3-E1, MG63, SaOS-2, ROS17/28 or primary isolated osteoblasts, all belong to osteoblasts, and they have been used as classic cell models for detection of osteoblastic differentiation stimulated by biomaterials [85–89]. Generally, osteoblasts tend to attach more and spread better on osteoinductive Ca-P, and their secretion of ALP, as well as some osteoblastic differentiation markers, are also promoted by these materials. For example, according to the microarray analysis, the phenotype of mouse MC3T3-E1 osteoblasts was significantly altered by Ca-P with varied phase composition, especially the increased ALP activity and mRNA expression of ALP, COL-I and OPN [80]. Many reports showed that Ca-P ceramics with different phase compositions changed the mRNA expression of ALP, COL-I or OCN in diverse kinds of osteoblasts [87–89].

#### Expressions of bone-related genes and proteins

As mentioned earlier, during the natural osteogenesis process, a lot of genes and proteins, especially bone-related genes, will be activated. These genes and proteins, therefore, have been considered as markers of osteogenesis and have provided significant evidences for material osteoinduction. These markers include BMP-2, BMP-4, BMP-7, Runx2/Cbfa1, Osterix, COL-I, OPN, OCN, BSP and ALP (Fig. 3).

BMPs are potent osteoinductive factors that have been evaluated extensively for both preclinical and clinical use. They can regulate the function and differentiation of cells involved in bone formation and bone healing [90]. The original discovery of BMP-2 as osteoinductive factors of DBM prompted researchers to study BMPs’ expression levels in ectopic bone formation induced by Ca-P [89, 91].

*In vivo*, gene expressions of BMP-4 were detected as early as the third day after the implantation of BCPs [92]. Eyckmans [91] found that the mRNA expression of BMP-2, -4, -6 and -7 in human periosteum-derived cells (hPDCs) increased over time, during bone formation process induced by Ca-P granules.

In an *in vivo* experiment, the immunohistochemical staining showed the highest expression of BMP-2 and OCN in BCP, corresponding to the stronger osteoinductive ability presented in this BCP group [8]. In the *in vitro* co-culture with MSCs, more osteoinductive Ca-P ceramics induced higher expression of BMP-2 and BMP-4 [83, 93].

Besides BMPs, researchers paid attention to lots of other bone specific genes and proteins which were also related to osteoinduction of biomaterials. For example, the mRNA expressions of COL-I, ALP was detected from the 3rd day to 24th week upon the implantation of BCP ceramics in the dorsal muscle of dogs [92]. The protein secretion of osteonectin (ON), OPN and OCN were also detected by immunohistochemical staining after intramuscular implantation of HA, BCP or TCP ceramics in rats [80].

All these bone-related markers are used to mark or predict the trend of osteogenesis by materials. For example, expression of COL-I and OCN genes in human bone marrow MSC (BMSCs) was higher on Ca-P surfaces than on tissue culture plastic [81]. Another report also showed that more osteoinductive materials contributed to a relative higher expression of many genes involved in bone matrix formation [94].

Altogether, these reports offered cellular and molecular evidence for Ca-P-stimulated ectopic bone formation or material osteoinduction. Moreover, based on these data and models, we can further design specific experiments in the future to get a more comprehensive understanding of this osteoinduction process, which is to unveil the cellular and molecular mechanism for osteoinduction of Ca-P.

## Mechanism of osteoinduction

### Cells and signals related to bone building and repair

**Figure 5.**
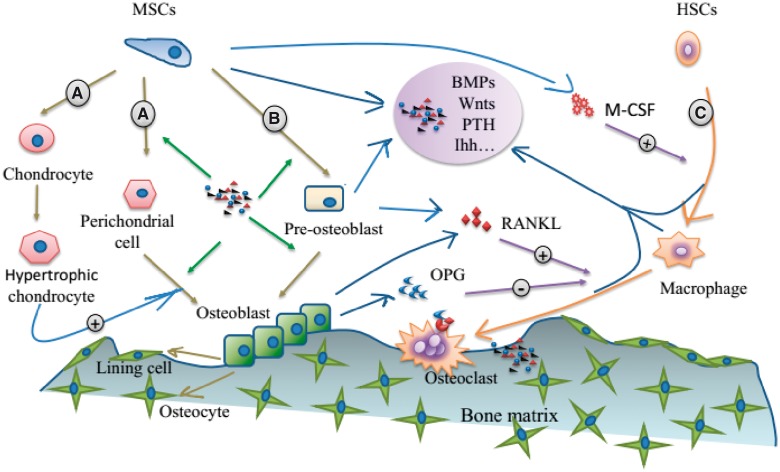
Cells and signals related to bone building and repair: (**A**) the osteoblastic differentiation in endochondral ossification, (**B**) the osteoblastic differentiation in intramembranous ossification and (**C**) osteoclast differentiation of monocytes in hematopoietic stem cells

As the ectopic bone formation induced by materials resembles the natural bone both in morphology and function, it is reasonable to search for cues from the cellular and molecular aspect involved in the building and repair of natural bone, for exploring the mechanism of material osteoinduction [10, 13, 72, 92]. The cells and signals related to bone building and repair are shown in Fig. 5.

The building of bone during vertebrate embryogenesis involves two linages of cells: osteoclast and osteoblast lineage cells. The balance between the production and function of osteoblasts and osteoclasts is crucial for bone homeostasis [95].

As the chief bone-making cells, osteoblasts can differentiate from MSCs through two processes: endochondral or intramembranous ossification (marked by A and B in Fig. 5). The two processes both start with the aggregation and condensation of MSCs. Then, with adequate vascularization, intramembranous ossification happens as MSCs directly differentiate into osteoblasts. By contrast, during endochondral ossification, MSCs differentiate into perichondrial cells and chondrocytes, the latter then undergo hypertrophy, which then triggers the differentiation of perichondrial cells to osteoblasts [95]. Later, accompanied with the invasion of blood vessels, the chondrocytes produced cartilage would gradually be replaced by bone [96]. Thus, it seems that no matter through which process, the formation of bone is closely related to angiogenesis. Accordingly, every step along osteogenic differentiation from MSCs in either way is regulated by various molecules, including morphogens, hormones, growth factors, cytokines, matrix proteins, transcription factors and their co-regulatory proteins. These molecules work as signaling pathways, such as BMP signaling [97], Hedgehog signaling [98], Notch signaling [99], Wnt signaling [100] and FGF signaling [101]. These signaling pathways may interact with each other in orchestrating bone formation or bone repair [102, 103].

For the osteoclast differentiation (marked C in Fig. 5), i.e. osteoclastogenesis, it includes the transformation of mononuclear hematopoietic stem cells (HSCs) to bone marrow-derived macrophages, and the further forming of osteoclasts. These transformations are mainly mediated by macrophage colony-stimulating factor (MCSF), receptor activator of NF-κB (RANK), receptor activator of NF-kB ligand (RANKL), with osteoprotegerin (OPG) as a natural decoy receptor for RANKL [104].

These elements are further regulated by various kinds of biological factors from diverse types of cells in interrelated ways. For instance, the tumor necrosis factor alpha (TNF-α) and interleukin-6 (IL-6), secreted by macrophages in response to innate inflammation, can activate T-cell to express soluble RANKL [105] and up-regulate the RANK on the surface of osteoclast precursors, thus inducing osteoclastogenesis [106–109]. Some cytokines secreted by endothelial cells, such as IL-4 and IL-13, can stimulate stromal cells to express OPG to antagonize osteoclast differentiation [110].

Thus, altogether, the osteoblast differentiation and osteoclastogenesis are regulated by various cytokines of different cells, which make a complicated crosstalk between these cells from different systems, which at least involve the bone, vascular system and immune system [109]. Therefore, in regard to ectopic bone formation induced by Ca-P, studying these cells and related processes might unveil the mechanism in cellular and molecular level.

Many studies have revealed the biological process of Ca-P-induced bone formation, as mentioned earlier. These studies have not only offered enormous insight into these cells and events but also raised questions for further understanding. As various kinds of cells and events were observed before the emerging of osteoblast, hereby, the first question arose for unlocking the mechanism is that: what kind of cells is the precursor for the osteoblast? Then, as the osteoinductivity of Ca-P is intrinsic, which means their inherent properties could somehow interact with cells and other biological factors *in vivo* to induce the ectopic bone formation, thus the second critical question for unveiling the mechanism is: how do the materials trigger the accumulation and subsequent osteogenic differentiation of those precursors?

### Cell origin of osteoblasts

For the first question, as it has been described before, the presentation of MSCs and their differentiation into osteoblasts have been demonstrated in the Ca-P-induced osteoinduction, which undoubtedly indicate that these MSCs are precursors of the osteoblasts. However, the following question remains: where do these MSCs come from to rest in Ca-P materials within the ectopic site?

Previously, it was observed that lots of fibrous connective tissue and capillaries invaded into the internal pores of Ca-P ceramics, where the novel bone formation begins [13]. Furthermore, it was found that some of the polymorphic cells that aggregated in association with capillaries in the vicinity of the ceramic surface could be positively stained for the ALP [72]. These indicate that the capillaries and the connective tissue that grow into the internal pores of Ca-P ceramics may provide the MSCs for further differentiation [111]. Similarly, Ripamonti [79] observed that, before and during bone formation, laminin staining (for vascular endothelial cells) was localized around capillaries in the vicinity of the ceramic, as well as around individual cells that seemed to migrate out of the vascular compartment. In all, osteoblastic differentiation from MSCs seems to be first observed near capillaries, as well as the internal surface of ceramics. Therefore, pericytes or endothelial cells associated with the capillaries were proposed to be the origin of those cells that aggregate and undergo osteoblastic differentiation in the vicinity of ceramics. This was further supported by the fact that ectopic bone formation induced by Ca-P biomaterials occurs almost via the intramembranous ossification [16].

Endothelial cells compose the inner lining of blood vessels and lymphatic vessels [112]. They are similar to epithelial cells, which can undergo epithelial–mesenchymal transition (EMT) to participate in physiological processes such as wound healing. This means endothelial cells can also transform into mesenchymal cells via a process known as endothelial–mesenchymal transition (EndMT) [113]. Specifically, a recent study has found that in a pathological heterotopic ossification, the vascular endothelial cells differentiated into chondrocytes and osteoblasts through an intermediate of MSCs which were generated by EndMT; and this differentiation was mediated by local inflammatory signals and/or other changes in the tissue microenvironment [114]. This work makes it reasonable that in the Ca-P material induced heterotopic bone formation, the endothelial cells may contribute to the origin of osteoblast by undergoing EndMT. Interestingly, a stem cell population with myoblastic and endothelial characteristics was found within the striated muscular tissue to support the continuous cell differentiation and replacement [115, 116]. This population of stem cells was defined as myoendothelial stem cells, and these myoendothelial stem cells were proposed as the cells that can differentiate into osteoblasts when in contact with osteoinductive biomaterials by Ripamonti [117].

With regard to pericytes, they are ubiquitous subendothelial cells which reside in vessels ranging in size from the microvasculature to the aorta [116]. Basically, pericytes serve as perivascular progenitor cells, which play an important role during capillogenesis, as they invest budding endothelial sprouts and control the proliferation and differentiation of endothelial cells [118]. Recently, it was found that pericytes are mesenchymal cells that have been described to have stem-like properties and differentiate into other cell types [119, 120]. However, others suggested pericytes may arise from EndMT [112, 121, 122]. Specifically, pericytes could express ALP [123, 124], and it has been suggested that they are a supplementary source of osteoblasts in periosteal osteogenesis [125]. Hence, pericytes were suspected to contribute to the process of bone formation at the surface of an osteoinductive material [16].

As a matter of fact, MSCs could originate from several other resources, such as bone marrow. In one of our group’s earlier works, we have observed that MSCs originating from bone marrow presented in the pores of Ca-P by immunohistochemical staining [126]. This was further confirmed by a recent study, which demonstrated the homing of BMSCs to non-osseous sites for ectopic bone formation, induced by osteoinductive Ca-P [127].

Fibroblasts, another kind of MSCs, may also contribute to the origin of osteoblasts, since they are present in the connective tissue that grows into the pores of ceramics. Furthermore, our *in vitro* study, which investigated the osteoinductive effect of Ca-P on C2C12 fibroblasts [128], supported this theory.

### Signals and their origin for initiating the osteoblastic differentiation in osteoinduction of Ca-P

It is generally accepted that MSCs are the origin of OB in the ectopic bone formation induced by materials. However, what triggers the MSCs’ accumulation on the material surface and the subsequent osteogenic differentiation remains unclear.

#### Adsorption or concentration of proteins that initiate the osteoinduction

Many studies have demonstrated that Ca-P showed a high affinity for proteins by providing higher amount of surface with high surface energy, as well as by providing Ca^2+^ positively charged binding site for negatively charged proteins. Thus, protein adsorption would be the first event upon the implantation of Ca-P. And since several physical or chemical features, such as topographical features (e.g. roughness, micro-porosity) of Ca-P strongly affect the amount and specificity of adsorbed proteins, just as they have been shown to affect the osteoinduction of Ca-P, it is reasonable to consider this process may be relevant to the osteoinduction, or even be the very reason of osteoinduction by Ca-P.

##### Adsorption of BMPs on Ca-P

In the exploration for the mechanism of osteoinduction by Ca-P biomaterials, it has long been suspected that endogenous BMPs might be the very biological reason [13], due to the early works of Urist [129].

This hypothesis was supported by our *in vitro* study, which showed that Ca-P ceramics have a strong ability to absorb bovine BMPs. Further supporting evidence was obtained from the following *in vivo* studies, in which the immunohistochemical BMPs staining before bone formation in the ceramics was detected after 30 days upon implantation in pigs, in addition to enhanced bone formation in Ca-P ceramics by bovine BMPs [11]. Moreover, it was found that inhibition of BMPs by a monoclonal antibody resulted in reduced osteoinductivity of the Ca-P [130]. Therefore, the importance of BMPs in osteoinduction of Ca-P is undeniable. In addition, this was shared by Ripamonti et al. [131], as they also detected BMP-3 and BMP-7 on the interface of tissue-HA substrate, where bone was observed after implantation in the muscle of primates.

Later, since the deposited apatite layer after implantation facilitated the adhesion of proteins such as BMPs, De Groot [132] proposed that the dissolution and deposition of a biological apatite layer on Ca-P surface leads to the co-precipitation of BMPs, to the sufficient concentration to trigger the osteoinduction.

Although the theory concerning the adsorbed or co-precipitated BMPs contribute to the osteoinductivity of Ca-P is reasonable, there are still apparent differences between osteoinduction by BMPs and that by Ca-P biomaterials. For instance, Ca-P-induced ectopic bone formation usually occurs via intramembranous ossification [21, 133, 134], while BMPs induced ectopic bone formation is mostly through endochondral ossification [135].

##### Other proteins adsorbed on Ca-P

Besides BMPs, other proteins are also absorbed by Ca-P. Among them, cell-adhesive proteins, such as fibronectin and vitronectin, can bind with specific integrin on the cytomembrane to generate anchors for cells, as well as initiate the cell–ECM interactions, which have been shown to influence different kinds of cell behavior, such as adhesion, proliferation and even osteogenic differentiation [136, 137]. This specificity of binding and the related modulation of cell behavior were dominated by the different types of integrins, and their association with the cellular signaling network to initiate downstream signaling cascades, such as the focal adhesion kinase (FAK), protein kinase C (PKC) and mitogen-activated protein kinase (MAPK) pathways [138].

In this aspect, Matsuura et al. [139] suggested that the RGD domains of fibronectin and vitronectin on Ca-P surfaces play major roles in the spreading of osteoblasts, thus contributing to the osteoconductivity of the surfaces. Later, Kilpadi proved that hydroxylapatite binds more cell adhesive proteins such as fibronectin and vitronectin from the serum than do two commonly used hard-tissue materials, namely pure titanium and stainless steel. In turn, this increased protein adsorption would lead to better binding of integrins α5β1 and α5β3, as well as osteoblast precursor cells [140].

In Marino’s [141] study, which cultured human adipose-derived stem cells on TCP surfaces in the absence of supplements, the presence of phosphorylated-FAK and evidence of differentiation were observed. Subsequently, in the study of Salasznyk et al. [142], human BMSCs on COL-I and vitronectin coated surfaces showed osteoblastic differentiation via the phosphorylation of FAK, which resulted in the activation of Runx2. Therefore, it is plausible that the Ca-P adsorbed cell-adhesive proteins, such as vitronectin, may modulate osteoblastic differentiation through the subsequent phosphorylation of FAK and activation of the extracellular signal-regulated kinase 1/2 (ERK1/2) pathway. Recently, it was found that α2β1 integrin and MAPK/ERK signaling pathways were involved in the regulation of osteoconduction for TCP by the BMP-2 autocrine loop [143].

Moreover, based on the overlap between the integrins and BMP-2 receptors [144], an integrin-BMP/Smad signaling pathway was suggested to promote bone regeneration for nanofibrous HA/chitosan scaffolds, in which the HA accounts for cell adhesion or spreading by influencing integrin-mediated signaling cascades and the BMP pathway in BMSCs [145].

#### Osteoinduction due to interaction between MSCs and Ca-P

Since several types of cells would co-exist with the Ca-P in the *in vivo* environment after implantation, new theory for the osteoinductive mechanism was brought up, which implied it was the materials triggered secretion of factors in these cells, rather than accumulation of factors from body fluid, that lead to bone formation. This theory can be divided into two parts according to the cells (MSCs or HSC-derived cells) that were directly affected by Ca-P. For this part, we discuss the MSCs which are directly stimulated by Ca-P to undergo osteoblastic differentiation through their own secreted factors or other changes, as well as how the Ca-P triggered this effect.

##### Ca-P-stimulated signaling molecules in MSCs

In searching for the critical molecules that initiate the osteoblastic differentiation of stem cells stimulated by Ca-P materials, once again BMPs drew the attention of many researchers because of their potent osteoinductive capacity. Previously, though immunohistochemical stains of BMPs have been observed [126, 131], it was still not sure where these BMPs came from, or whether they were adsorbed or secreted, and if so, by what kind of cell**s**. Later, in a clinically relevant model of the osteoinduction process, which required the existence of Ca-P and BMP/Wnt signaling, the activation of the BMP/Smad signaling pathway in hPDCs was evidenced by immunohistochemistry stains for p-Smad 1/5/8 positive cells [91]. Recently, to avoid other cells and their cytokines within the *in vivo* environment, we conducted an *in vitro* experiment by simply culturing the BMSCs on HA and BCP ceramics in the absence of additional osteogenic factors [93]. These two ceramics were shown to be osteoinductive by a previous *in vivo* study [77], with BCP showing higher osteoinductivity. By comparing the osteoblastic differentiation status and gene expression of related molecules in BMP/Smads signaling, we suggested that Ca-P ceramics may initiate the osteoblastic differentiation of MSCs by stimulating the autocrine of BMP-2 in MSCs, as well as by up-regulating other relevant molecules in the BMP/Smads pathway [93]. Since BMP-2 could induce expression of other BMPs by an autocrine pathway [90, 146], we further suspected that this material-stimulated BMP-2 up-regulation may result in a cascade of amplification for BMP signaling. This *in vitro* study was in line with our *in vivo* study [8], as in which BMP-2 secretion was proved to be stimulated by osteoinductive Ca-P, and the highest was correlated with the best ectopic bone formation. Barradas’s [83] recent *in vitro* study, which compared HA and β-TCP ceramics, showed a similar result with regards to BMP-2 expression. Thus, the osteoinductive Ca-P stimulated BMP-2 autocrine loop in MSCs may be the very factor which initiates the osteoblast differentiation. The involvement of BMP-2 was further supported by an *in vivo* inhibitory study, which demonstrated that the blocking of calcium channel and osteoclastogenesis resulted in down-regulation of BMP-2 and limited bone formation [147].

Besides the BMP autocrine signaling loop, other signaling pathways that are involved in bone development or repair might also be related to the osteoinduction process triggered by Ca-P. Through *in vitro* co-culture of MSCs with TCP or HA ceramics, Barradas et al. [83] found MSCs cultured on β-TCP showed more pronounced attachment and spreading than that on HA. Subsequently, using DNA microarray analysis, they also found that the MSCs were induced to express G-protein coupled receptor (GPCR) 5 A and regulator of G-protein signaling 2 by β-TCP [83]. Therefore, they proposed that these genes, which were related to the protein kinase A and GPCR signaling pathways, might serve as the prediction of the earliest response of MSC to bone-inducing ceramics [83]. Recently, a study demonstrated that secretion of IL-1α in BMSCs was stimulated by the nanohydroxyapatite (nHAp) within some kind of composite scaffolds, and it was through an autocrine/paracrine way [148]. Moreover, the activity of IL-1α could be mediated by the producing of IL1R2 when BMSCs interacted with nHAp [148].

The adenosine signaling pathway is also involved. For instance, Shih [149] proved that by the release of phosphate, which participated in the synthesis of ATP in mitochondria of cells, Ca-P-bearing matrices could induce stem cells to differentiate along osteoblastic linage via the adenosine signaling.

Using *in vivo* study, Eyckmans [150] mapped the Ca-P-activated gene network by ectopic implantation of constructs consisting of hPDCs, with HA/collagen scaffolds (Ca-P-rich matrix, CPRM), or decalcified scaffolds (Ca-P-depleted matrix, CPDM). The result revealed that both constructs stimulated a similar gene expression cascade upon subcutaneous implantation, yet the gene expression dynamics was slower in CPDM scaffolds than in osteoinductive CPRM scaffolds [150]. The variation in gene expression dynamics was supposed to be related to the hub genes’ differential activation, as well as the molecular signaling pathways associated with bone development (TGF-β, β-catenin, BMP, EGF and ERK signaling), calcium signaling (CREB) and inflammation (TNF-α, NF-κB and IL-6) [150].

##### How does Ca-P stimulate MSC to secrete these signaling molecules?

Though the expression of BMP-2 or other signaling molecules in MSCs stimulated by Ca-P were confirmed, it was still unknown how this happens. Just like Klar [147] has correlated BMP-2 expression and bone formation with calcium ions, this signaling also originates from the physical and chemical or structural nature of Ca-P. The Ca^2+^, ${\text{PO}}_{4}^{3}$^−^ ions and concavity or geometry are suspected as essential factors of Ca-P to achieve osteoinduction according to their related signal pathways in MSCs.

###### Calcium ions (Ca^2+^ ions)

The Ca^2+^ ion is the most universal carrier of biological signals, which modulates cell life from its origin at fertilization to its end in the apoptotic process [151]. Firstly, it was suggested that extracellular Ca^2+^ gradients could work as a chemotactic signaling for homing of bone marrow progenitor cells or pre-osteoblast to the site of bone resorption [27, 28, 152, 153]. Then, higher concentration of Ca^2+^ could also stimulate these progenitor cells’ maturation into cells that produce new bone [28, 153]. Thus, it is reasonable to suspect that the Ca^2+^ ions, released from the dynamic dissolution of Ca-P materials, may recruit MSCs chemotactically, and stimulate their osteogenic differentiation in a concentration-dependent way.

Previously, Jung et al. studied the effects of HA released Ca^2+^ on osteoblastic differentiation of MC3T3-E1 cells. They suggested that the released Ca^2+^ may enter cells through calcium channels and calcium sensing receptors (CaSR), and activate the CaMK2a/CAM pathway [154]. This may finally modulate the osteoblastic differentiation via the CREB and/or the extracellular signal-regulated kinase 1/2 (ERK1/2) pathway [155]. Later, Barradas et al. demonstrated that a high extracellular concentration of Ca^2+^ enhanced proliferation and morphological changes in hMSCs, accompanied with up-regulation of BMP-2 expression. However, the interference with a series of proteins involved in Ca^2+^ sensing showed that it was not the CaSR, but rather type L voltage-gated calcium channels were involved in mediating the signaling pathway between extracellular Ca^2+^ and BMP-2 expression. Furthermore, their microarray analysis indicated that MEK1/2 activity was essential for the effect of calcium, thus they came to propose that the internalization of Ca, probably by ion channels, would activate PKC, EK1/2, ERK1/2 pathways in sequence, and at last enter the nucleus to up-regulate the BMP-2 expression via Fos expression and activator protein 1 (AP-1) formation [156]. This study is in line with an *in vivo* study, which showed that blocking of Ca ions channel would result in down-regulation of BMP-2 expression and limited bone formation [147].

There is also another theory about the osteoinductive mechanism of Ca-P that involves Ca^2+^. Syed-Picard [157] demonstrated that amorphous calcium phosphate alters cellular functions and 3D spatial tissue differentiation patterns by increasing local calcium concentration, which in turn modulated the connexin 43 mediated gap junctions between cells.

###### Phosphate ions (${\text{PO}}_{4}^{3}$^−^ ions)

For the released phosphate, previous work by Beck [158, 159] showed that a higher concentration of phosphate can induce mineralization-associated genes, such as matrix glaprotein (MGP) and OPN, in MC3T3-E1 cells through the activation of ERK1/2- and PKC-dependent pathways, rather than the p38-dependent pathway. Nevertheless, the study of Khoshniat et al. [160] showed that calcium is required for phosphate-dependent ERK1/2 phosphorylation to regulate MGP/OPN expression, since this effect may originate from extracellular-related effects of Ca-P that are dependent on the integrity of lipid rafts. Recently, Shih [149] proved that released phosphate from Ca-P-bearing matrices could enter the cells via a phosphate transporter called solute carrier family 20 member1 (SLC20α1). Then, the internalized phosphate participated in the synthesis of ATP, which would promote the osteoblastic differentiation of hMSCs as an autocrine/paracrine signaling molecule. Moreover, the osteoblastic differentiation would not happen if the SLC20α1 was perturbed, due to the decreased intra-mitochondrial phosphate and ATP synthesis [149]. Therefore, it seems that ${\text{PO}}_{4}^{3}$^−^ released by Ca-P may work in diverse means which involve different signal pathways.

Particularly, Chai et al. [161] showed both ions, administrated individually or in combination with calcium, consistently up-regulated BMP-2 expression for human periosteum-derived cells in a dose-dependent manner. Barrada’s recent work and our *in vitro* study, which both used Ca-P ceramics instead of ions, showed the similar result with regards to BMP-2 expression [83, 93]. Thus, both ions released by Ca-P may be involved in triggering the BMP-2 autocrine signaling loop, even though they may work through different pathways.

Altogether, these studies showed that either ion can effectively promote the osteoblastic differentiation of progenitor cells. Alternatively, their combination may be more osteoinductive, for this combination may form other elements, such as the Ca-P precipitates [160], which may enhance the osteogenesis by other means that individual ions could not achieve.

###### Physical or structural characteristics

Apart from the adsorption of related proteins (integrin or growth factors) and the chemical influence of Ca^2+^ and ${\text{PO}}_{4}^{3}$^−^ ions, the Ca-P could also directly influences cells through its physical or structural characteristics, such as the grain size, surface structure or geometry, which may also contribute to the osteoinduction of Ca-P.

Regarding molecular mechanism, it was reported that these physical or structural factors worked mainly in a mechanically sensitive way through those molecules which participated in cytoskeletal tension generation, including actin, myosin II, Rho, ROCK and the Rho modulator [162]. A recent study showed that geometric features increase actomyosin contractility promote osteogenesis; its microarray analysis and pathway inhibition studies suggested that contractile cells promoted osteogenesis by enhancing c-Jun N-terminal kinase and ERK1/2 activation in conjunction with elevated wingless-type (Wnt) signaling [163].

Besides integrin and associated cytoskeletal filaments, cells may also utilize other cell surface molecules to sense or transduce mechanical stress, such as cadherins and selectins that form intercellular junctional complexes or the stress-sensitive ion channels on the cell surface [164–167]. The concavity of porous ceramics may generate shear stress forces of the *in vivo* fluid, which work through mechanosensitive ion channels to direct MSCs differentiation [168, 169]. Furthermore, the concavity would work as a protective environment to accumulate the released Ca^2+^ and ${\text{PO}}_{4}^{3}$^−^ ions, thus recruit and stimulate the differentiation of MSCs [29, 170, 171].

#### Osteoinduction due to interaction between Ca-P and other cells

Except MSCs, there are other cells participating in the host response to materials, and these cells may be more important for osteoinduction than MSCs. What’s more, the interaction between these cells via their secreted cytokines factors may be even more important.

##### Interaction between Ca-P and inflammation-associated cells

Actually, the specific inflammatory response of tissues to the osteoinductive ceramics is an important factor that renders a material osteoinductivity [16, 134, 172, 173]. As described previously, macrophages/monocytes are involved in the inflammation process, as well as in the bone remodeling or repair process. This, along with the appearance of these cells in the ectopic bone formation induced by Ca-P, makes a link between the inflammatory response and osteoinduction process of Ca-P. Certainly, the osteoclasts and their resorption activity would also be involved since they are derived from these macrophages/monocytes.

###### Interaction between Ca-P and macrophages/monocyte

The inflammation process started from the wound by injury. However, with Ca-P existed by the implantation, the consistently available Ca^2+^ ions would cause the circulating monocytes to fuse into macrophages. Then the macrophages, under certain stimulation, may secrete cytokines that could induce the osteoblast differentiation of available MSC cells. For instance, De Bruijn et al. [174] showed that macrophages in response to micro-rough surfaced HA, which were osteoinductive, particularly produced more prostaglandin E2 (PGE2) than that of smooth HA, and this PGE2 was shown to be chemotactic for hMSCs and could stimulate their osteoblastic differentiation.

Recently, in the study of Chen, it was found that macrophage phenotype switched to M2 extreme in response to β-TCP extracts, and this was related to the activation of CaSR; BMP-2 was also significantly up-regulated by the β-TCP stimulation, which indicates that macrophages may participate in the β-TCP-stimulated osteogenesis. Moreover, when macrophage-conditioned β-TCP extracts were applied to BMSCs, the osteogenic differentiation of BMSCs was significantly enhanced, indicating the important role of macrophages in biomaterial-induced osteogenesis [175]. Not only the ions, but also the microparticles from the degraded Ca-P, can influence the behavior of those monocytes or macrophages, in addition to factors such as TNF-α, IL-6 and IL-10 [134, 176, 177]. Actually, harnessing the power of macrophages/monocytes to get enhanced bone tissue engineering has been put forward by Dong et al. [178]. They suggested that several osteotropic factors (such as IL-1 b and IL-4) could be produced by macrophages/monocytes, and that these cytokines played constructive roles in bone development and repair. The significant role of macrophages/monocytes in osteoinduction could also be illustrated by the implantation of respective scaffolds in nude mouse, which demonstrated that BCP associated with total bone marrow, which contained MSCs and macrophages/monocytes, consistently improved osteoinduction than BCP associated with pure human stromal cells [179].

###### Interaction between Ca-P and osteoclasts

The continuous presence of Ca^2+^ released by the Ca-P, as well as the presence of incompletely dissolved Ca-P, could promote the macrophages to undergo further transformation into osteoclasts, i.e. osteoclastogenesis [180, 181]. Once osteoclasts attached, the Ca-P ceramic is resorbed subjacent to the ruffled border of the cell by acid hydrolysis. The resorption by osteoclasts results in concavity, either as lacunae, grooves and/or pits, as the osteoclasts trek across the surface of the Ca-P, which in turn works as micro-topographic cues to affect the MSCs’ differentiation [169]. For the macro-porous Ca-P-based constructs, which primarily/only possessed a macro-topographical geometric surface, this resorption by osteoclasts to create concavities was proved to be essential for them to achieve ‘intrinsic’ osteoinductivity [117]. This process induced the micro- and macro-topographical geometric surface patterning alterations, as Ripamonti [117] proposed based on their study which utilized Zoledronate Zometa to inhibit this effect of osteoclasts.

Additionally, osteoclastogenesis and the osteoclastic priming of the macro-porous surfaces may also result in nanotopography, which have been proved to be capable of controlling stem cell differentiation [45, 182]. Moreover, Ca^2+^ and ${\text{PO}}_{4}^{3}$^−^ ions released by the resorption activity of osteoclasts also contribute to the osteogenic differentiation and subsequent bone formation [183], as Ca^2+^ and ${\text{PO}}_{4}^{3}$^−^ ions accumulated in the concavity would reach a higher concentration.

For the micro-porous Ca-P, which already possessed micro or even nanoscale features before implantation, the osteoclastogenesis and the osteoclast resorption was not irrelevant or less important than their osteoinduction. Recently, it was showed that the physical–chemical nature of the micro-porous Ca-P, especially the surface architecture, could affect the osteoclastogenesis [184] and osteoclast resorption [185], which in turn would influence the osteoinduction of this Ca-P. In the latter study, the TCPs, which exhibited osteoinductivity with submicron-scale surface structure, induced osteoclastogenesis and ectopic bone formation in a process that was blocked by monocyte/macrophage depletion, in comparison with TCPb, which were not osteoinductive with micron-scale surface architecture [185]. By utilizing the same pair of materials, the authors also demonstrated that the osteoclast resorption of Ca-P was also controlled by tuning of surface architecture, with only the submicro-structured TCPs showed to be resorbed by osteoclast. Particularly, they showed that the foreign body giant cells could not resorb either TCP material, suggesting that osteoclast specific machinery is necessary to resorb TCP [185].

Thus, it could be seen that the specific inflammatory response to Ca-P, which involved the macrophages/monocyte or osteoclasts, would help to create or enhance several factors which could mediate the ectopic bone formation, including soluble cytokines (BMP-2, PGE-2, TNF-α, IL-6, IL-10), insoluble topographic features (concavities) and the accompanied higher concentration of Ca^2+^ and ${\text{PO}}_{4}^{3}$^−^ ions, or cytokines.

Additionally, besides macrophages/monocytes, other kind of cells, such as T cells and B cells in the immune system, may also play a role in this osteoinduction process. In the Nse-BMP4 mice, the lack of mature B and T lymphocytes resulted in smaller spreading and overall amount of heterotopic ossification, which suggested that the adaptive immune system plays a role in spreading of heterotopic ossification [186]. Recently, developments in osteoimmunology have revealed the vital role of immune cells in regulating bone dynamics. Chen et al. [187–189] have suggested that neglecting the importance of the immune response may explain the inconsistencies between *in vitro* and *in vivo* conditions, and they proposed osteoimmunomodulation in recognition of the importance of the immune response during biomaterial-mediated osteogenesis.

##### Interaction between Ca-P and cells associated with angiogenesis

As described in the cell origin section of this review, the ectopic bone formation by Ca-P always needs the preformation of blood vessels, which hints the importance of angiogenesis to the osteogenesis induced by Ca-P. Actually, the cells responsible for the angiogenesis are endothelial cells and pericytes, which may also be able to transition into osteoblasts under the mediation of inflammation, as described previously. Moreover, these cells can express cytokines such as BMP-2 and BMP-7 [190], and their expression has been shown to be markedly up-regulated in response to inflammatory stresses [191, 192]. In fact, BMPs are cable of not only regulating osteogenesis but also angiogenesis [193]. To add a further level of complexity, BMPs are capable of regulating EndMT [114], which would provide MSCs for further differentiation. Thus, the role of angiogenesis in the osteoinduction of Ca-P may come through several ways. One is through their secreted cytokines, such as BMPs, to recruit and induce MSCs to undergo osteoblastic differentiation. A second way is by providing more endothelial cells and pericytes to transform into MSCs, which finally differentiate into osteoblasts under the induction of materials or cytokines. The final way is to serve as a conduit for other osteoprogenitor cells, such as BMSCs. These aspects are only in accordance with how the angiogenesis contribute to the initiation of osteoinduction. As for the whole osteoinduction process, it should not be forgotten that the new blood vessels are essential for providing oxygen and nutrients to support the growing bone.

Furthermore, recent studies have found that the angiogenesis in ectopic bone formation of Ca-P is also influenced by materials. Our recent *in vivo* study showed that the more resolvable TCP resulted in more blood vessels in the ectopic site than did other HA or BCP ceramics, which indicate the effect of Ca^2+^ ions in angiogenesis [34]. Actually, Ca^2+^ mediated signaling is important in angiogenesis [194, 195], and extracellular Ca was suggested to be a key factor responsible for the angiogenic responses of bone marrow progenitor cells [196]. Other factors, such as concavity, have also been suggested to trigger rapid angiogenesis through the Ca^2+^ ions retained within it. Further, the angiogenesis could work with mechanical distortion to trigger the invasion of MSCs (myoblastic/myoendothelial and/or endothelial, pericytic) to rest in the concavity [170]. Hereby, within this protective microenvironment, MSCs may response to the accumulated ions or cytokines, or to topographic features to undergo differentiation [169].

Together, as one of the pre-requisites for osteoinduction of Ca-P [134], the angiogenesis showed to work with Ca-P to promote osteogenesis. However, even with sufficient angiogenesis, osteoinduction of Ca-P will not definitely happen. This may be due to the role of inflammation in mediating the angiogenesis and the EndMT, as well as the secretion of osteogenic factors from the endothelial cells. Thus, as the inflammation also interacted with Ca-P, the proper combination of Ca-P-triggered inflammation with angiogenesis might be the reason for osteoinduction.

### Summary for the mechanism of material osteoinduction

**Figure 6.**
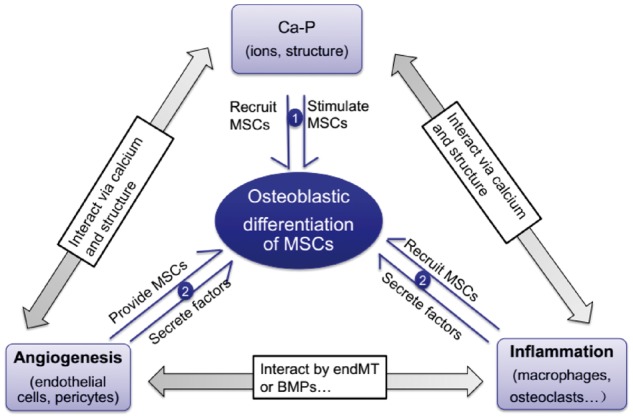
Hypothesis for the osteoinductive mechanism of Ca-P: ① represents the direct effect of Ca-P (as the changed form) to stimulate the osteoblastic differentiation of MSCs and ② represents Ca-P interacting with the inflammatory cells to trigger the osteoblastic differentiation of MSCs. This may involve the angiogenesis, which could interact with both the Ca-P and inflammation

In summary, the mechanism for osteoinduction of Ca-P may be very complicated, owing to the involvement of various kinds of cells, molecules, as well as biological progresses with crosslinked interactions. As we have depicted the existing theories concerning the adsorption of proteins, the interaction between Ca-P materials and different types of cells, and none of them have been isolated as the specific reason for the osteoinduction of Ca-P, we therefore propose a hybrid hypothesis for the osteoinduction mechanism of Ca-P, which involves not only the bone system, but also the vascular and immune systems (shown in Fig. 6).

Based on the previous studies and theories, it can be seen that micro-porous Ca-P may work through soluble factors (such as Ca^2+^ and ${\text{PO}}_{4}^{3}$^−^ ions), and/or insoluble factors (such as their micro or even nanoscale topographic features) to interact with various kinds of proteins and cells, as well as mediate their interaction. Therefore, a summarized hypothesis for the osteoinduction mechanism of micro-porous Ca-P is formed: firstly, the injury caused invading of the inflammatory factors and inflammatory cells. Secondly, the micro-porous Ca-P work through soluble factors (such as Ca^2+^ and ${\text{PO}}_{4}^{3}$^−^ ions), and/or insoluble factors (such as their micro or even nanoscale topographic features), or even by interacting with these inflammatory factors and cells, which results in adsorption or concentration of related proteins (osteogenic growth factors, cell adhesive proteins and inflammatory factors), as well as the recruitment of various kinds of progenitor cells (monocytes, MSC, endothelial cells, pericytes). This process may be accompanied by the dynamical dissolution/precipitation of Ca-P, and co-precipitation of proteins to form the bone apatite-like layer on the Ca-P surface. Then, there are two pathways that may result in the osteoinduction: the first is that Ca-P (as the changed form) directly stimulates the osteoblastic differentiation of MSCs (marked 1 in Fig. 6). The second is by Ca-P interacting with the inflammatory cells (monocytes, macrophages, osteoclast), which result in more osteoinductive surface features and higher concentration of Ca^2+^ and ${\text{PO}}_{4}^{3}$^−^ ions, as well as osteogenic cytokines. Finally, these factors trigger the MSCs’ differentiation (marked 2 in Fig. 6). The latter pathway may involve the angiogenesis, which could interact with both the Ca-P and inflammatory response to contribute to osteoinduction by providing more osteogenic factors and more MSCs (marked 2 in Fig. 6).

## Perspectives: from osteoinductive materials to tissue-inducing materials

Since the discovery of Ca-P osteoinduction, extensive studies have been performed to deepen our understanding of this fantastic phenomenon, as well as its possible mechanism. The key inspiration from this is that we can endow the materials with the biofunction of inducing tissue regeneration. The realization of this idea would thoroughly change the concept of biomaterials. Although there are still many unclear secrets hiding behind the material osteoinduction, it guides us to explore revolutionary change in material design. Based on this concept of material osteoinduction, extensive attempts to design tissue-inducing materials have been performed [42, 163, 197–202]. These frontier studies include the design of material characteristics to change cell fate, such as the osteogenesis or chondrogenesis differentiation, and the phenotype and multipotency maintenance of MSCs [46]. In some pioneer studies, chondrogenesis, neuranagenesis and angiogenesis are reported [34, 197–202].

In summary, current biomaterials science and engineering is undergoing revolutionary change. Tissue-inducing biomaterials are becoming an important direction and frontier for biomaterials science and industry.
